# Evaluation of the Hypothalamus‐Hypophysis‐Adrenal Axis in Male Rats Programmed by Gestational Protein Restriction

**DOI:** 10.1002/cbf.70123

**Published:** 2025-10-04

**Authors:** Vinicius Schiavinatto Mariano, José Antonio Rocha Gontijo, Patrícia Aline Boer

**Affiliations:** ^1^ Department of Internal Medicine, School of Medicine State University of Campinas Campinas São Paulo Brazil; ^2^ Fetal Programming and Hydroelectrolyte Metabolism Laboratory, Internal Medicine Department Faculty of Medical Sciences at State University of Campinas Campinas São Paulo Brazil; ^3^ Internal Medicine Department, School of Medicine State University of Campinas Campinas São Paulo Brazil

**Keywords:** catecholamines, fetal programming, hypothalamic–pituitary–adrenal axis, protein‐restricted diet, stress

## Abstract

Diet manipulations during the gestation of animal models, in this case, the lipoprotein diet, mimic the alterations related to low birth weight, providing studies of the mechanisms involved in chronic disease development in later life. Our research group identified in adult male rats submitted to gestational protein restriction, increased anxiety‐like behavior, basal plasmatic corticosterone (CORT) and catecholamines elevation, and decrease of hippocampal glucocorticoid receptors (GRs), indicating dysfunction of the stress response, which is related to the sympathetic‐adrenomedullary system and the hypothalamic–pituitary–adrenal (HPA) axis alterations. Not only insults during gestation but also maternal care behavior during breastfeeding can modulate the HPA axis of the offspring, influencing its activity in adulthood. Thus, we evaluated maternal care behavior and morphological and functional parameters of the adrenal and pituitary glands in gestational protein‐restricted male rats to elucidate mechanisms that can trigger these possible alterations. Mated Wistar rats were submitted to a normal‐protein diet (NP group; 17% protein) or a low‐protein diet (LP group; 6% protein) throughout pregnancy. From the day of birth until weaning, the maternal care behavior parameters were evaluated, and at the 16th week of age, plasma, adrenal, and pituitary glands were collected for hormonal analysis by LC‐MS/MS, western blot, and immunohistochemistry. LP offspring animals showed low birth weight and recovered at weaning, indicating the effect of catch‐up growth. No difference in maternal care behavior was found between the groups, suggesting that maternal care may not influence the decrease of hippocampal GR in LP offspring. The plasma levels of 11‐dehydrocorticosterone (11‐DHC) in 21PND and 16‐week‐old LP offspring decreased, whereas the plasma levels of CORT and 11‐DHC of 8‐week‐old LP offspring increased. GR and mineralocorticoid receptors, essential to glucocorticoids' practical actions, were increased in the pituitary and adrenal glands in LP 16‐week‐old animals, indicating possible negative feedback. However, the 98.8% increase in CRH receptor and 63.3% ACTH in the pituitary of the LP offspring indicates failure of this feedback at the pituitary level. The morphometric analysis of the LP 16‐week‐old animal's adrenal gland showed an increase in medullary area, accompanied by an increase of 39.67% in NeuN, indicating an increase in medullary cellularity and an increase of 168.77% in PCNA, suggesting a cell proliferation under the demand of adrenal hyperactivity. In addition, an increase of 5‐HT1A receptor (48.69%) in the LP adrenal gland, which is associated with inhibitory catecholamine secretion, and an increase of immunostaining of 5‐HT1A and 5‐HT2A receptors differently within the pituitary lobes, suggesting modulation of the HPA axis at the pituitary level through the serotonergic innervation from hypothalamic CRH neurons. Gestation protein restriction results in adult rat offspring, morphological and functional changes in the adrenal glands, and hormonal modulations associated with stress responsively and adrenergic hyperactivity. These alterations could participate in the genesis and maintenance of hypertension in this model.

## Summary

- LP offspring animals showed low birth weight and recovered at weaning, indicating the effect of catch‐up growth.
- No difference in maternal care behavior was found between the groups, suggesting that maternal care may not influence the decrease of hippocampal GR in LP offspring.
- The plasma levels of 11‐dehydrocorticosterone (11‐DHC) in 21PND and 16‐week‐old LP offspring decreased, whereas the plasma levels of CORT and 11‐DHC of 8‐week‐old LP offspring increased.
- GR and mineralocorticoid receptors, essential to glucocorticoids' practical actions, were increased in the pituitary and adrenal glands in LP 16‐week‐old animals, indicating possible negative feedback.
- However, the 98.8% increase in CRH receptor and 63.3% ACTH in the pituitary of the LP offspring indicates failure of this feedback at the pituitary level.
- The morphometric analysis of the LP 16‐week‐old animal's adrenal gland showed an increase in medullary area, accompanied by a rise of 39.67% in NeuN, indicating an increase in medullary cellularity and an increase of 168.77% in PCNA.
- Finally, an increase of 5‐HT1A receptor (48.69%) in the LP adrenal gland, which is associated with inhibitory catecholamine secretion, and an increase of immunostaining of 5‐HT1A and 5‐HT2A receptors differently within the pituitary lobes.

**Abbreviations**
11‐DHC: 11‐dehydrocorticosterone
11β‐HSD1: 11beta‐hydroxysteroid dehydrogenase type 1
11β‐HSD2: 11beta‐hydroxysteroid dehydrogenase type 2
5‐HT: 5‐hydroxytryptamine (serotonin)
5‐HT1A: type‐1 5‐HT receptor, 5‐hydroxytryptamine receptor or serotonin receptor
5‐HT2A: type‐2 5‐HT receptor, 5‐hydroxytryptamine receptor or serotonin receptor
ACTH: adrenocorticotropic hormone
AGD: anogenital distance
ANG‐II: angiotensin‐II
ANOVA: analysis of variance
AT1: angiotensin receptor type 1
AT2: angiotensin receptor type 2
AVP: arginine vasopressin
CEMIB/UNICAMP: Unicamp Multidisciplinary Center for Biological Research
CEUA/Unicamp: State Campinas University Institutional Ethics Committee
CNS: central nervous system
CORT: corticosterone
CRF: corticotropin‐releasing factor
CRF1: type‐1 CRF receptor
CRH: corticotropin‐releasing hormone
CRHR: CRH receptor (CRHR1 and CRHR2)
DOHaD: Developmental Origins of Health and Disease
DPN: post‐natal day
EDTA: ethylenediamine tetraacetic acid
Epi: epinephrine
GC: glucocorticoid
GR: glucocorticoid receptor
GZ: granular zone
HPA: hypothalamic–pituitary–adrenal
LP: low‐protein diet
MR: mineralocorticoid receptor
MSH: melanocyte stimulating hormone
NE: norepinephrine
NeuN: neuronal nuclear protein
NP: normal‐protein diet
PCNA: proliferating cell nuclear antigen
PNMT: phenyl ethanolamine N‐methyltransferase
POMC: proopiomelanocortin
RP: Rathke's pouch
SAS: sympathetic‐adrenomedullary system
SERT: serotonin transporters
TH: tyrosine hydroxylases
WHO: World Health Organization

## 1 Introduction

Gestational malnutrition, a pressing public health issue, is implicated in infant morbidity and mortality. The World Health Organization (WHO) reports that 15%–20% of all newborns have low birthweight, totaling about 20 million low‐weight newborns worldwide [1]. The embryo‐fetal exposure to maternal undernutrition leads to permanent alterations of the structure and physiology of specific organs, predisposing low birth weight, directly related and generally irreversible to metabolic and endocrine disease, as well as behavioral and cognitive changes in adulthood. This theory, known as fetal programming, underscores the long‐term impact of malnutrition during critical periods of development [2, 3, 4, 5]. The concept of a “thrifty phenotype” [4], an economic phenotype adapted to its demands, plays a significant role in fetal programming. It further underscores the long‐term implications of fetal programming. The fetal response to adversities during gestation, conceptualized as a predictive adaptive response, proposes that the developing organism adapts to the environment not only to obtain an immediate advantage but also to predict the environmental incompatibility, with a higher risk of developing chronic non‐communicable diseases, such as hypertension, type 2 diabetes, and obesity [6, 7, 8]. These adaptive effects can occur in different windows of development, whether during preconception, gestation, the first years of life, or even adolescence. The concept of Developmental Origins of Health and Disease (DOHaD) is crucial in understanding these adaptations and their implications on adult health throughout life [6]. Several studies relate low birthweight to the development of chronic diseases in adulthood, and studies in our laboratory have consistently demonstrated the interference of environmental factors, mainly gestational malnutrition and smoking stress, on the structural and neurotransmitter development of regions of the central nervous system (CNS) involved in the behavioral control of rodents [9, 10, 11, 12, 13]. An English survey conducted with a population of men and women between 1911 and 1930 showed that the mortality rate from cardiovascular diseases was higher in individuals who were born with low birth weight [14]. In addition, it is well established that high blood pressure, cardiovascular diseases, and low birth weight have also been associated with elevated cortisol levels in adulthood, a marker of stress linked to cardiovascular diseases. Phillips et al. [15] showed higher fasting cortisol concentrations associated with cardiovascular disease in adults with low birthweight in England and Australia. The neuro‐humoral stress response occurs via the hypothalamic–pituitary–adrenal (HPA) axis, which consists of a complex set of interactions promoting the plasma release of glucocorticoids (GCs) [16]. More studies are crucial to understanding the physiological mechanisms that involve the stress response and its impact on fetal development.

The adrenal gland is the primary source of corticosteroids: cortisol, corticosterone (CORT), and aldosterone in rodents. The synthesis and secretion of corticosteroids are governed by the anterior pituitary release of adrenocorticotropic hormone (ACTH), which in turn is controlled by the hypothalamic secretion of corticotropin‐releasing hormone (CRH) and arginine vasopressin (AVP) [17]. In physiological conditions, the concentration of GC in the embryo‐fetal plasma is kept lower than that of the mother's levels due to the action of the placental enzyme 11β‐hydroxysteroid dehydrogenase type 2 (11β‐HSD2) [18]. This enzyme converts bioactive cortisol and CORT into their inactive metabolites, cortisone and 11‐dehydrocorticosterone (11‐DHC). However, it has been reported that stress, drug use, malnutrition, and poor nutrition during pregnancy promote a decrease in the concentration and activity of placental 11β‐HSD2, consequently increasing fetal exposure to GC. This increased exposure can lead to intrauterine growth restriction, low birth weight [19, 20, 21], and its association with cardiovascular and metabolic diseases in adulthood. Our research in the Lab has unveiled the profound impact of fetal programming by maternal undernutrition on the development of the sympathetic nervous system and the modulation of norepinephrine (NE) turnover in several organs [13, 22, 23, 24]. These studies consistently demonstrate the changes in the structural and neurotransmitter content in areas of the CNS involved in the behavioral sympathetic neural control of rodents [11, 12, 13]. In the maternal protein‐restricted model, the pups show accelerated weight gain, known as catch‐up growth, in the mothers returning to a standard normal‐protein diet (NP) intake after birth [9, 10, 24]. This catch‐up growth, which compensates for the offspring's low birth weight, is associated with environmental incompatibility due to the adaptive predictive response discussed above; in other terms, this effect may have advantages for short‐term survival but at a high biological cost [6, 7, 8, 25, 26]. In the protein‐restricted model, behavioral changes and HPA axis structural and functional abnormalities, characterized by a 28% reduction in the nephron number, decreased urinary sodium excretion, and increased sympathetic renal nerve activity, are related to elevated blood pressure [9, 10, 13, 20, 22, 23]. Custódio et al. [23] demonstrated increased renal and plasma catecholamines in low‐protein diet (LP) offspring at 16 weeks compared to age‐matched NP progeny. When bilateral renal denervation was performed in these offspring, a decreased renal content of catecholamines associated with enhanced renal sodium excretion and reduced BP was observed. These findings, even if indirect, suggest an increased sympathetic nerve activity in the LP progeny since denervation prevents the onset of arterial hypertension in LP animals, pointing towards potential benefits for future research and interventions. In a study by Cardoso et al. [22], the intracerebroventricular (i.c.v.) epinephrine (Epi) administration promotes increased natriuresis and a reduction in BP in LP animals by increased proximal and post‐proximal sodium excretion, with no changes in glomerular filtration. The natriuretic effect was attenuated significantly by α1‐adrenergic receptor antagonist simultaneous administration—conversely, the i.c.v. administration of α2‐adrenergic receptor antagonists potentiated the natriuretic capacity of i.c.v. adrenergic stimulation. When LP animals were pretreated with an α2‐adrenergic antagonist, urinary sodium excretion normalized, as well as a decrease in BP [9, 20, 22, 23].

Thus, in the maternal protein‐restricted model, in addition to the involvement of the HPA axis in the programming of the animals, our prior results indicate the effective participation of the sympathetic nervous system [23].

The adrenal gland is a crucial part of the HPA axis and plays a vital role in the body's physiological response to stress through the sympathetic‐adrenomedullary system (SAS), which affects plasma levels of catecholamines. Our results indicate that the LP offspring exhibit an increased state of stress responsiveness, suggesting an exaggerated activation of the stress axis. Neuroendocrine feedback mechanisms are crucial for regulating the stress response and function at multiple levels of the HPA axis, including the pituitary gland. To explore the potential effects of gestational low‐protein exposure on the HPA and SAS‐related stress response pathways, this study aims to evaluate serum concentrations of adrenal hormones at various developmental stages. Furthermore, at 16 weeks of age, we assessed the morphology and protein expression related to the functional regulation of the hypothalamus, as well as the adrenal and pituitary gland structures in male offspring born to mothers subjected to gestational protein restriction.

## 2 Materials and Methods

### 2.1 Animals

All the experimental procedures were approved by the institutional Ethics Committee on the Use of Animals at the State University of Campinas (CEUA protocol 4811‐1). Wistar rats were purchased from the Multidisciplinary Center for Biological Research (CEMIB/UNICAMP) and kept under a standard 12 h light/dark cycle with a controlled temperature at 22°C with libitum access to food and water. Female Wistar rats (0.250–0.300 g) were mated during the dark cycle, and the pregnancy was confirmed by a vaginal smear on the following morning (0 days of pregnancy). Once pregnancy was confirmed, the dams were singly housed, and an isocaloric and normosodic NP (17% of casein) or LP (6% of casein) chow was offered throughout pregnancy. The dietetic composition of both chows is available in Table S1. The dams were weighed weekly, and the food intake was evaluated. At birth, the offspring were weighed, the anogenital distance (AGD) measured, and both experimental groups returned to a normoprotein standard chow diet. Also, the number of pups was adjusted to eight for each litter to normalize the food access for the pups. The food intake and weight of dams were measured until weaning. The weaning occurred at PND21 of offspring, in which the males were allocated to cages with ad libitum access to food and water, and the food intake and weight were evaluated until adulthood. Males' offspring from both NP and LP groups were euthanized in the 16th week of age using isoflurane as the anesthetic (4% in O_2_ vaporizer). Trunk blood was collected in a 1% heparinized microtube to obtain the plasma sample, and the pituitary and adrenal glands were collected. For histological analysis, the 16‐week‐old male offspring were euthanized and transcardially perfused (pressure 80–100 mmHg) with 1% heparinized saline following paraformaldehyde (PFA) 4% diluted in phosphate buffer 0.1 M pH 7.4. The pituitary and adrenal glands were collected and post‐fixed for 4–5 h in the same PFA solution, washed in PBS 1X, and stored in EtOH 70% until analysis.

### 2.2 Steroid Hormone Levels Measurement by MS‐LC/MS

The stress‐related steroid hormones CORT, 11‐DHC, and aldosterone levels were measured in plasma samples using a high‐resolution multiple reaction monitoring method (MRMHR). The samples were extracted and purified using solid phase extraction. After the purification, the samples were dried and resuspended in MeOH: Milli‐Q water (7:3) for injection in the LC‐MS/MS system. The LC‐MS/MS analysis was performed as described (2) utilizing MS TripleTOF 5600+ (SCIEX) coupled to LC Nexera (Shimadzu Corp.).

### 2.3 Immunohistochemistry

The process of paraffin‐embedding for the pituitary glands in rats was adapted from a study by Cao et al. The dehydration of the glands was done using a series of decreasing EtOH solutions, consisting of EtOH 95% for 15 min once and three consecutive times in absolute EtOH for 5 min. Next, the glands were cleared in xylene for 5 min and embedded in Paraplast at 60°C for 6–8 min, three times. For the adrenal gland, the following protocol was used (put for the adrenal gland). Coronal sections (5 μm) of both the pituitary and adrenal glands were obtained using a Leica RM2245 microtome and mounted on silane‐coated slides. To perform immunohistochemistry, the slides were first deparaffinized, hydrated, and washed in TBS/TBS‐T and then subjected to antigen retrieval in citrate buffer at pH 6.0 using a steamer for 0.5–1 h. A nonimmune serum goat or rabbit (Table S2) was used to block the tissue for 2 h to prevent unspecific antibody binding. The primary antibody was diluted in bovine serum albumin 3%, and the slides were incubated overnight at 4°C. The concentrations of each primary antibody used are detailed in Table S2. The slides were then washed, and endogenous peroxidase was blocked using a 1:10 methanol/H_2_O_2_ solution for 10 min and incubated with a secondary antibody with HRP (Table S2) for 2 h in RT. The slides were washed, and the ImmPACT DAB (Vector) was used as a substrate to promote staining, according to the manufacturer. We opted to counterstain only for cytoplasmatic/membrane proteins with Harris' hematoxylin. The slides were dehydrated, diaphanized, and mounted with Entellan for analysis.

### 2.4 Micrographs Acquirements and Morphometric Analysis

The micrographs were obtained in the Olympus BX51 microscope equipped with a DP71 camera using OLYMPUS CellSens Dimensions 1.15 software. For staining quantification of the pituitary gland, micrographs were obtained per animal: anterior (*n* = 15), intermediate (*n* = 4), and posterior (*n* = 3) pituitary, for each group. Then, each region was selected using the region of interest (ROI) tool, and the percentage of area stained was obtained. Four seriated slices were selected for morphometric analysis of the adrenal gland in the central adrenal gland area. Each area analyzed (total, cortex, and medulla; *n* = 5 each group) was selected by the ROI tool, and the area was expressed in μm^2^.

### 2.5 Western Blot

Both the adrenal and pituitary gland and the hypothalamus were homogenized using Polytron in lysis buffer containing EDTA 10 mM, Trizma base 100 mM, sodium pyrophosphate 10 nM, sodium fluoride 100 mM, sodium orthovanadate, phenylmethylsulfonyl fluoride 2 mM (diluted in absolute ethanol), aprotinin 0.1 mg/mL, Triton X‐100. The homogenates were centrifuged at 12,000 rpm at 4°C, and the supernatants of each sample were collected. The total protein concentration was determined by the biuret method and treated in a Laemmli buffer with 200 mM of dithiothreitol, heated at 100°C for 5 min, and applied in the polyacrylamide gel for SDS‐PAGE running. Initially, the gel was submitted to a 60 V current during the stacking running, and after the running, it was maintained at 120 V for 2 h. Then, the gel was coupled to the protein transfer system to transfer to the nitrocellulose membrane. The efficiency of protein transfer was confirmed by Ponceau S staining. For the western blot procedure, the membranes were washed in TBS‐T for 10 min three times (standard wash procedure in this protocol) and blocked in 5% nonfat milk for 2 h in RT. The membranes were washed and incubated with the primary antibodies overnight at 4°C in agitation. The primary antibodies are utilized, and their concentration is provided in Table S2. After incubation, the membranes were washed and incubated with conjugated HRP secondary antibodies for 2 h at RT. The protein bands were detected using Super Signal West Pico (Thermo Fisher) according to the manufacturer's protocol. The membrane was photo‐documented using GeneGnome (Syngene), and the band quantification was performed using ImageJ.

### 2.6 Data Presentation and Statistical Analysis

All data were reported as mean ± standard deviation (SD) of the mean. Data obtained over time were analyzed using a one‐way analysis of variance (ANOVA). When one‐way ANOVA analysis indicated statistical differences between groups, Bonferroni's contrast test performed post hoc comparisons between means. Comparisons between two groups were performed using a two‐way repeated measure ANOVA, in which the first factor was protein content in a dam's diet, and the second factor was time. When the interaction was significant, mean values were compared using Tukey's post hoc analysis. Student's *t*‐tests were used to evaluate studies involving only two independent samples, within or between groups. Welch's test was used to correct situations characterized by heteroscedasticity (different variances between groups). An animal's survival lifetime was assessed using Mantel–Cox and Gehan–Breslow–Wilcoxon tests. GraphPad Prism 5.00 was used for data analysis (GraphPad et al., USA). The level of significance was set at *p* ≤ 0.05.

## 3 Results

### 3.1 Gestational LP Diet Decreases Weight at Birth and Alters AGD in Rats

Regarding maternal parameters, LP did not affect the weight gain of dams (*F* = 0.2; *p* = 0.9) and food intake during gestation (*F* = 0.15; *p* = 0.8) compared with the NP group (Figure S1A,B; Table S3). At birth, gestational LP decreased the birth weight (*p* < 0.0001) in both male and female LP offspring compared with the NP group (Table 1; Figure S2A,B), and no differences were found in the number of total, female, and male pups born between the groups (Figure S2E–G). Also, the AGD was increased in LP males but not in LP females, compared with the sex‐matched controls (Table 1; Figure S2C,D). Despite being low‐weight at birth, no differences in the LP and NP offspring's weight and food intake were found from the 1st to 16th week of age (Figure S3A,B).

**Table 1:** Weight and anogenital distance (AGD) at the birth of offspring.

	Group	Mean ± SD	*N*	*p*‐value
Offspring birth weight (g)	NP male	6.92 ± 0.58	83	< 0.0001
	LP male	5.98 ± 0.54	85	
	NP female	6.19 ± 0.35	51	< 0.0001
	LP female	5.65 ± 0.65	67	
AGD (mm)	NP male	1.88 ± 0.17	67	0.0024
	LP male	1.98 ± 0.2	85	
	NP female	0.94 ± 0.1	53	0.3
	LP female	0.93 ± 0.09	61	

*Note:* Student's *t*‐test.

### 3.2 Basal Plasmatic Steroid Hormones Levels and HPA Axis‐Related Proteins Are Altered in LP Offspring

**Figure 1:**
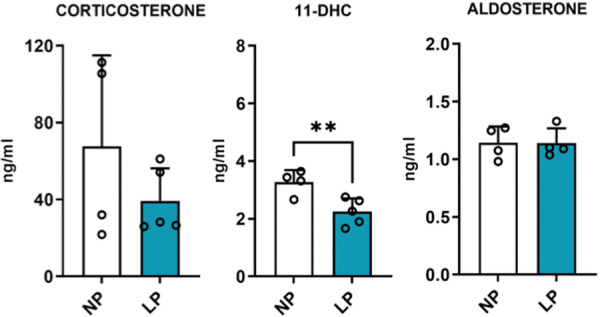
Basal plasmatic steroid hormone levels of both NP and LP offspring. Student's *t*‐test was used for statistical analysis. **p < 0.01. LP, low‐protein diet; NP, normal‐protein diet.

**Figure 2:**
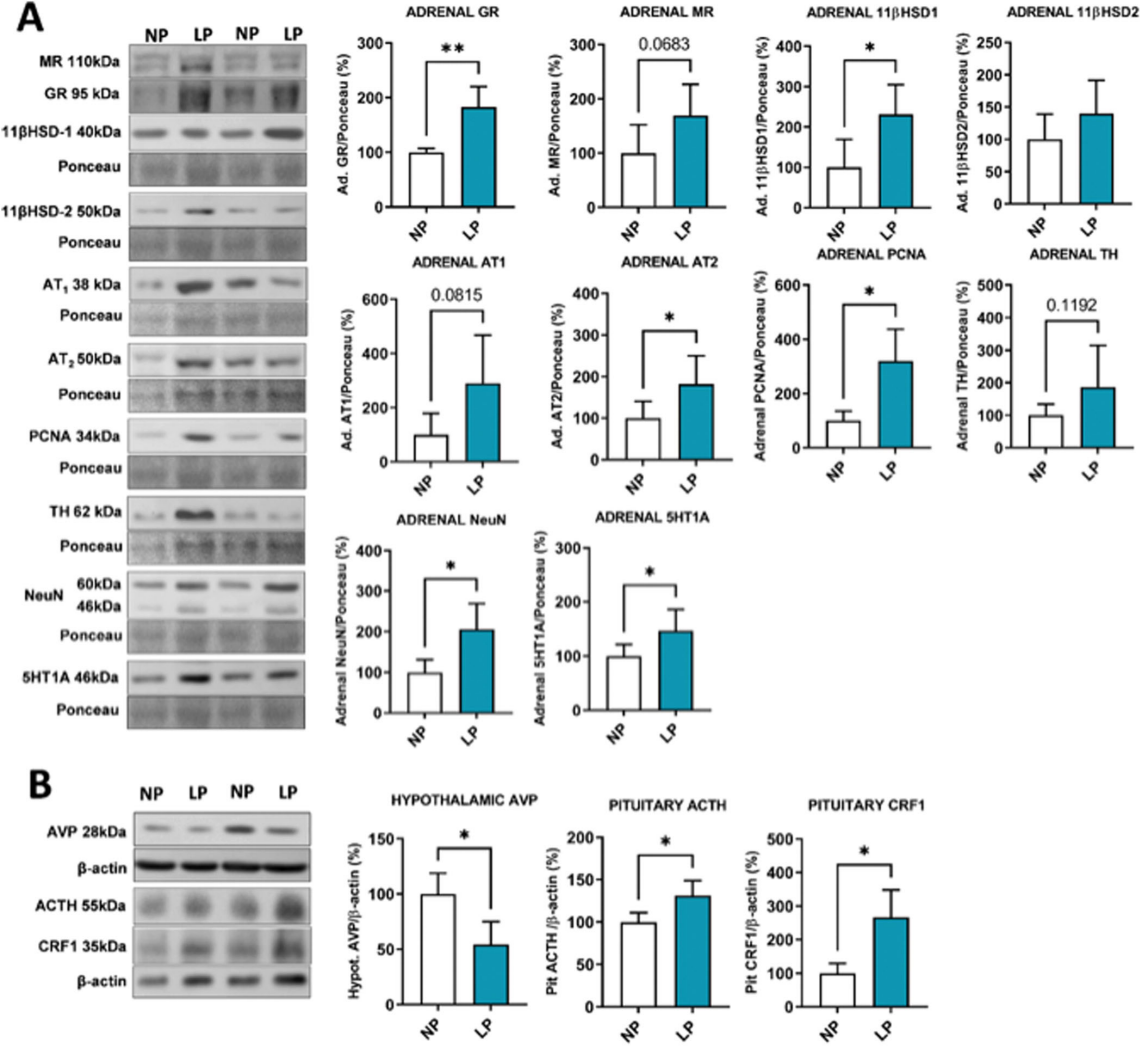
Western blot analysis of stress‐related proteins. (A) Western blot analysis of the adrenal gland and (B) the hypothalamus and pituitary of both NP and LP offspring. Student's *t*‐test was used for statistical analysis. *p < 0.05. LP, low‐protein diet; NP, normal‐protein diet.

The plasmatic basal steroid hormones were evaluated (Figure 1) and no differences were found between the levels of CORT (NP 67.6 ± 47.3, *n* = 4; LP 39.2 ± 16.9, *n* = 5; *p* = 0.1) and aldosterone (NP 1.14 ± 0.1, *n* = 4; LP 1.13 ± 0.1, *n* = 4; *p* = 0.4) in both experimental groups. Although a decrease (*p* = 0.005) in levels of the CORT metabolite, the 11‐DHC, was found in LP offspring (2.24 ± 0.4, *n* = 5) compared with controls (3.26 ± 0.4, *n* = 4). Despite no differences in plasmatic CORT basal levels, alterations in stress‐related protein content in LP offspring were found. In the adrenal glands, which are the principal source of steroid hormones, we found an increase of 83% of glucocorticoid receptor (GR) (NP ± 7%, LP ± 38%, *p* = 0.007) content and a tendency of increase of mineralocorticoid receptor (MR) (*p* = 0.06) in the LP offspring (Figure 2A). By western blot, we evaluated the content of 11beta‐hydroxysteroid dehydrogenase type 1 (11β‐HSD1) and 11β‐HSD2, and found an increase of 131% (*p* = 0.03) of 11β‐HSD1 in the adrenal gland of LP (± 74%) offspring compared with NP (± 69%), with no differences in 11β‐HSD2 content between groups (Figure 2A).

**Figure 3:**
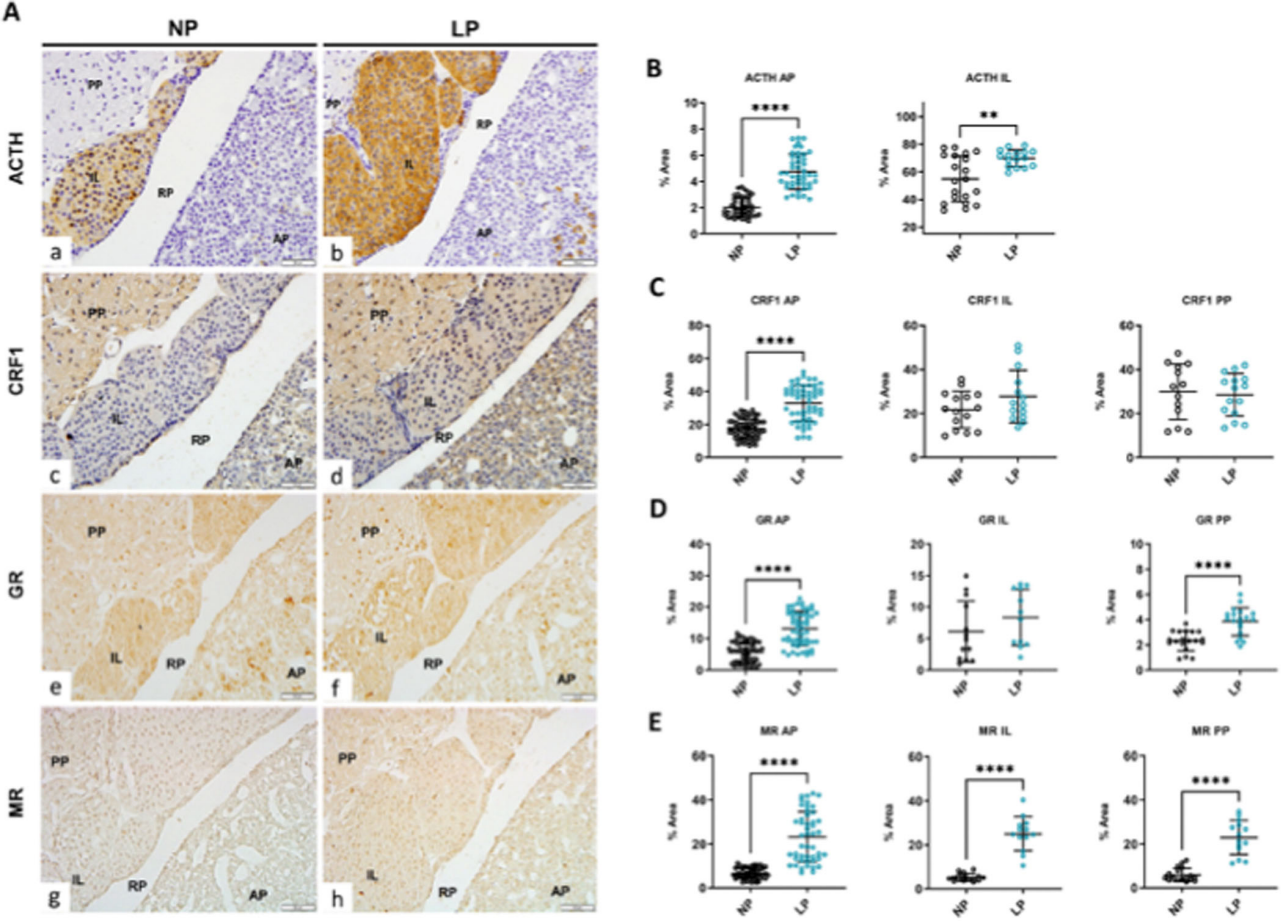
Immunohistochemistry of the pituitary gland of offspring. (A) Immunoperoxidase for ACTH (a and b), CRF1 (c and d), GR (e and f), and MR (g and h) of coronal sections of the pituitary gland of NP and LP offspring. Quantification of immunostaining of (B) ACTH in AP and IL, (C) CRF1, (D) GR, and (E) MR in all pituitary gland lobes of NP and LP offspring. Student's *t*‐test was used for statistical analysis. **p < 0.01, ****p < 0.0001. Scale bar: 50 μm. AP, anterior pituitary; IL, intermediate lobe; LP, low‐protein diet; NP, normal‐protein diet; PP, posterior pituitary; RP, Rathke's pouch.

At the pituitary level, an increase of 31% of ACTH content in LP offspring by western blot (*p* = 0.03) was found (Figure 2B). Immunohistochemistry analysis of the pituitary gland (Figure 3) revealed that the increase found by western blot of this protein occurred in a region‐specific manner. There is a predominated content of ACTH in IL, which is scattered throughout the lobe, and in isolated cells in AP, characteristic of corticotrophs, with no staining identified in PP (Figure 3A(a,b)). After staining quantification of pituitary regions, was found an increased (*p* = 0.0016) proopiomelanocortin (POMC)/ACTH immunomarker in the IL (NP 55 ± 16.5, *n* = 20; LP 70 ± 6, *n* = 16) and (*p* < 0.0001) in the AP (NP 2 ± 0.1, *n* = 45; LP 5 ± 0.2, *n* = 45) (Figure 3B) of the LP offspring compared to NP progeny. The impact of CRH on levels of POMC/ACTH was evaluated the content of CRHR1 in pituitary gland, which the content was increased in 166.92% (NP ± 29.6%, LP ± 80.9%, *p* = 0.01) in LP offspring pituitary gland (Figure 2B), and, by immunochemistry, this increase (< 0.0001) occurred only in AP (NP 18 ± 6, *n* = 72; LP 33 ± 11, *n* = 54) (Figure 3A(c,d) and 3C). Also, we evaluated the AVP content in the hypothalamus and we found a decrease of 45.6% (*p* = 0.01) in LP (± 21%) offspring, compared with NP (± 18.6%) (Figure 2B).

The current study also evaluates the GR content by immunostaining in the pituitary gland. A predominantly nuclear staining pattern of GR was found in all lobes and, in the AP, however, scattered cells were identified (Figure 3A(e,f)). These cells likely represent corticotrophs, as suggested by the similar staining pattern observed in ACTH immunostaining. The quantification of immunostaining content revealed an increase of GR in AP (NP 5.6 ± 3, *n* = 61; LP 13 ± 5, *n* = 55; *p* < 0.0001) and PP (NP 2.4 ± 0.8, *n* = 19; LP 3.9 ± 1.1, *n* = 19; *p* < 0.0001) of LP offspring (Figure 3D). The MR immunostaining shows predominantly nuclear staining pattern in all lobes (Figure 3A(g,h)), different from GR's, and we found an increase of content in AP (NP 6.6 ± 3, *n* = 54; LP 24 ± 12, *n* = 47; *p* < 0.0001), IL (NP 5.2 ± 2, *n* = 16; LP 25 ± 8, *n* = 13; *p* < 0.0001) and PP (NP 5.9 ± 3, *n* = 18; LP 23 ± 8, *n* = 14; *p* < 0.0001) in LP offspring (Figure 3E). Also, in adrenal gland, we evaluated the AT1 and AT2 content, and, despite no differences in aldosterone levels, we found an increase of 82% of AT2 (*p* = 0.02) in LP (± 68%) offspring compared with NP (± 41%), and a tendency of an increase in AT1 (*p* = 0.08) receptors in LP offspring (Figure 2A).

### 3.3 LP Diet Promotes Changes in Morphometric Parameters and Cellularity of Adrenal Gland in Offspring

**Figure 4:**
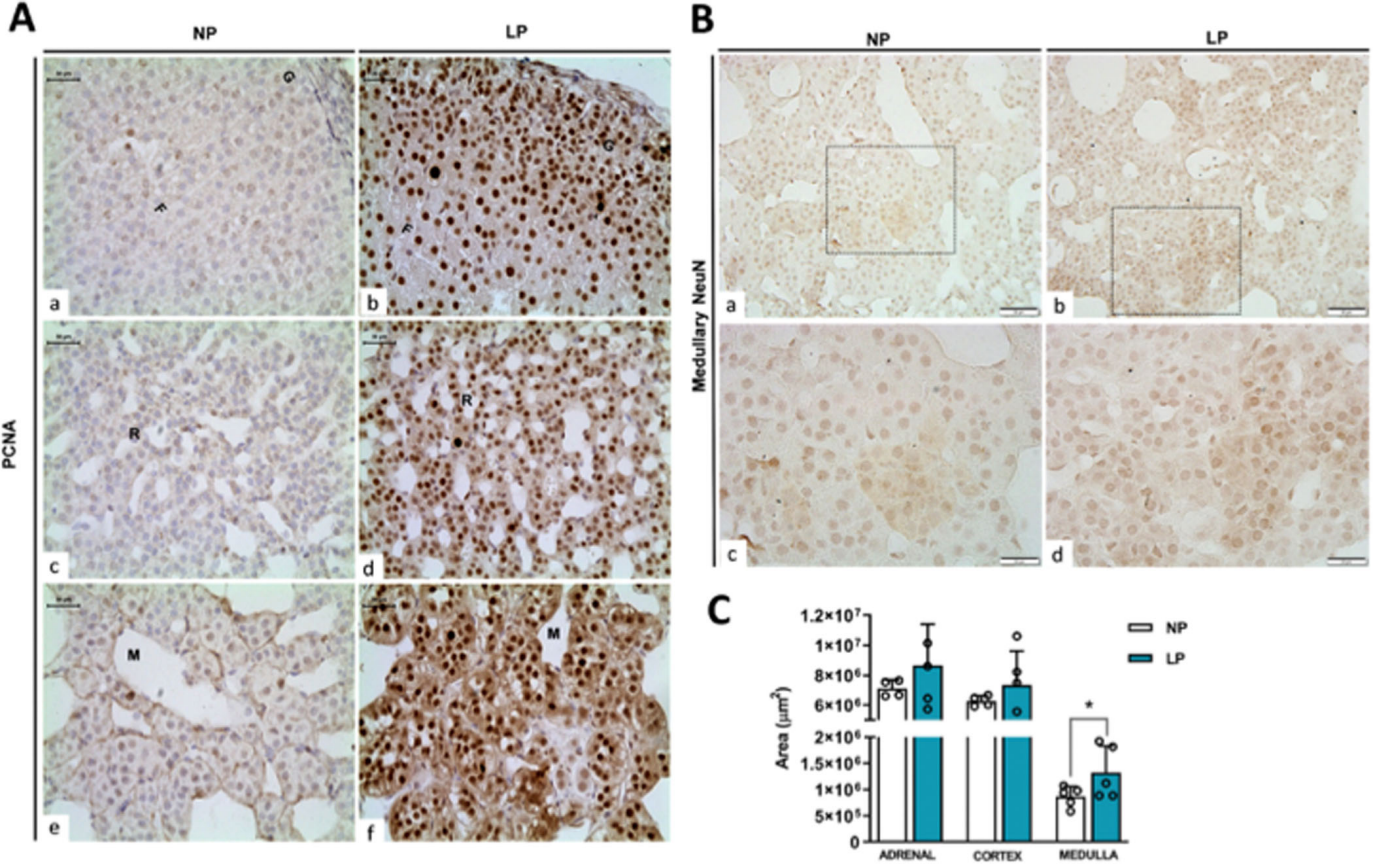
Morphometric and cellularity analysis of the adrenal gland of offspring. (A) Immunoperoxidase for PCNA of cortex (a–d) and medulla (e and f) of adrenal gland of NP and LP offspring. Scale bar: 30 μm. (B) Immunoperoxidase for medullary NeuN of the adrenal gland of NP and LP offspring. Scale bar: 50 μm (a and b), 20 μm (c and d). (C) Morphometric measure of total adrenal gland, cortex, and medulla area of NP and LP offspring. Student's *t*‐test was used for statistical analysis. *p < 0.05. F, fasciculata zone; G, glomerulosa zone; LP, low‐protein diet; M, medulla; NP, normal‐protein diet; R, reticulata zone.

In the adrenal gland, through morphometric analyses, we found no changes in total area (µm²) (NP: 7.118.472 ± 556.756, *n* = 4; LP: 8.668.308 ± 2.731.870, *n* = 5) and cortex area (NP: 6.269.771 ± 362.893, *n* = 4; LP: 7.343.577 ± 2284171, *n* = 5) of both experimental groups. Although, we found an increase (*p* = 0.047) in the medullary area of LP offspring (1324731 ± 505317, *n* = 5) compared with control (NP: 867027 ± 194157, *n* = 5), which represents an increase of 52.7% compared with control. To address possible parameters that corroborate these medullary area alterations in LP, we analyze the protein content of neuronal nuclear protein (NeuN), which identifies chromaffin cells with neuron phenotype (predominantly NE+ cells). We found an increase of 106% of NeuN (*p* = 0.01) in LP (± 63%) offspring adrenal gland compared with NP (± 31%) (Figure 2A), which was clearly evidenced by immunostaining (Figure 4B), but no differences were found in tyrosine hydroxylases (TH) content between groups (Figure 2A). Accompanied by these alterations, we found an increase (*p* = 0.02) of 220% of the cell mitotic marker proliferating cell nuclear antigen (PCNA) in LP (± 118%) offspring adrenal gland compared with NP (35%) (Figure 2A), which was evidenced in immunostaining that this increase occurred in all adrenal zones and medulla (Figure 4A).

**Figure 5:**
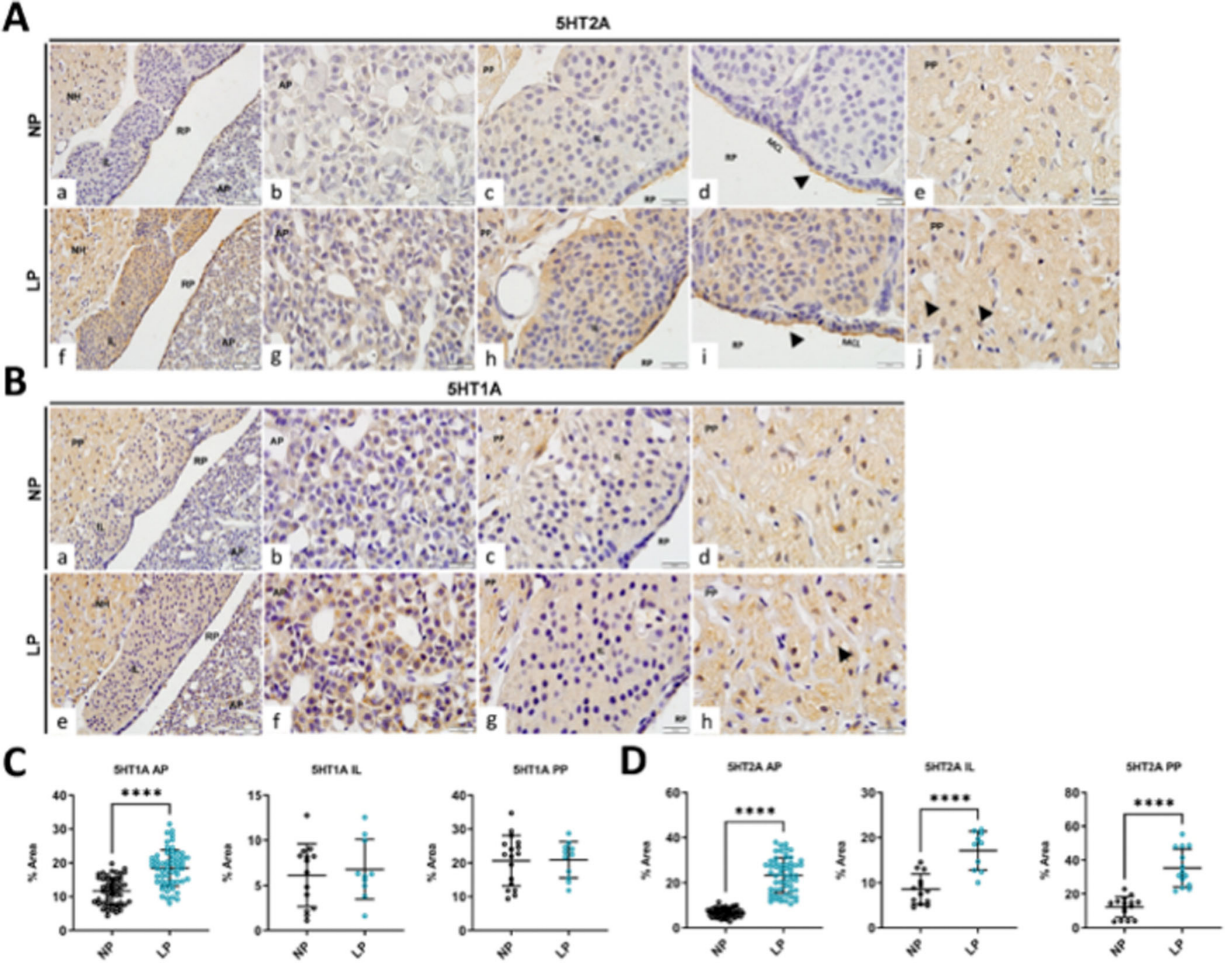
5‐HT receptors quantification in pituitary and adrenal glands of offspring. (A) Immunoperoxidase for 5‐HT2A receptor of the pituitary gland of NP and LP offsprings. Lobe's view (a and f), scale bar: 50 μm; amplified view of AP (b and g), of IL (c and h), of MCL (d and i), demonstrated the staining of ciliated cells (black arrow read), and PP (e and j) demonstrated evidenced Herring bodies (black arrow reads), scale bar: 20 μm. (B) Immunoperoxidase for the 5‐HT1A receptor of the pituitary gland of NP and LP offspring. Lobe's view (a and f), scale bar: 50 μm; amplified view of AP (b and g), of IL (c and h), and PP (e and j) demonstrated evidence of Herring bodies (black arrow read), scale bar: 20 μm. Quantification of immunostaining for (C) 5‐HT1A and (D) 5‐HT2A in all pituitary gland lobes of NP and LP offspring. Student's *t*‐test was used for statistical analysis. ****p < 0.0001. AP, anterior pituitary; IL, intermediate lobe; LP, low‐protein diet; MCL, marginal cell layer; NP, normal‐protein diet; PP, posterior pituitary; RP, Rathke's pouch.

The current study evaluates the 5‐HT1A and 5‐HT2A receptor contents in the adrenal and pituitary gland of NP and LP offspring. In the adrenal gland, we found an increase (*p* = 0.04) of 47% of 5‐HT1A content in LP (± 39%) offspring compared with control (NP ± 22%) (Figure 2A). In the pituitary gland, we performed immunostaining (Figure 5A,B) to identify these receptors, and both 5‐HT1A and 5‐HT2A were evidenced in a scattered pattern in all lobes. Interestingly, the staining evidenced the presence of Herring bodies in the PP (Figure 5A(i,j) and 5B(d–h), indicated by black arrows), and 5‐HT2A immunostaining highlighted the cilia of cells in the marginal cell layer of Rathke's pouch (RP). Comparing the experimental groups, we found an increase of 5‐HT1A content in AP of LP offspring (NP: 12 ± 0.5, *n* = 53; LP: 18 ± 0.6, *n* = 60; *p* ≤ 0.0001), with no differences in IL (NP: 6 ± 3, *n* = 14; LP: 7 ± 3, *n* = 10) and PP (NP: 21 ± 7, *n* = 17; LP: 21 ± 5, *n* = 11) (Figure 5B,C). In 5‐HT2A, we found an increase in AP (NP: 7 ± 2, *n* = 49; LP: 23 ± 8, *n* = 57; *p* ≤ 0.0001), IL (NP: 9 ± 4, *n* = 13; LP: 17 ± 4, *n* = 10; *p* ≤ 0.0001) and PP (NP: 12 ± 6, *n* = 15; LP: 35 ± 11, *n* = 14; *p* ≤ 0.0001) of LP offspring compared with NP (Figure 5A,D).

## 4 Discussion

Studies indicate that the intrauterine environment and early childhood are critical periods during which various factors—such as psychological, nutritional, and social stressors—can significantly impact individual development and have lasting effects on adulthood [27]. The maternal protein restriction model is frequently employed to explore the mechanisms of fetal programming and its consequences for adult health. Previous studies in our laboratory have identified various effects on offspring of rats, particularly in terms of cardiovascular and renal alterations, arterial hypertension [9, 10, 12, 13], behavioral changes, and dendritic morphology, as well as modifications in glucocorticoid and MR signaling in the hippocampus and the bed nucleus of the stria terminalis, which are associated with CNS and peripheral neural dysfunctions [11, 24, 28, 29]. This study examines potential disruptions in the HPA axis that may influence stress‐related behaviors and functional outcomes. Our findings confirm that gestational protein restriction leads to decreased birth weight in both male and female low‐protein progeny, validating this reduction as a significant programming marker. This decreased birth weight may arise from early cellular differentiation and is associated with reduced placental activity of 11β‐HSD2 and elevated fetal GC levels [11, 12, 29]. Notably, this reduction in birth weight did not correlate with decreased body mass gain or food intake in the pregnant [9, 10, 30, 31, 32] rats. Additionally, we confirmed an increase in AGD in both male and female LP offspring, consistent with previous findings [30]. Furthermore, as previously noted, the LP offspring exhibited recovery in body mass after the first week of life [9, 10, 11, 24], a phenomenon known as *catch‐up growth*, closely linked to programmed changes [6, 7, 8]. The current study observed a reduction in blood 11‐DHC levels associated with elevated basal aldosterone. However, no significant changes were noted in the hormonal stress response, including aldosterone, which was considerably diminished and subsequently restored after 21 DPN. Research indicates that the renin‐angiotensin‐aldosterone system (RAAS) plays a critical role in renal development during gestation and postpartum. This involvement primarily affects renal type 1 and type 2 angiotensin‐II receptors (AT1 and AT2) [33, 34], correlating with a lower number of nephrons at birth in LP compared to NP offspring. While previous findings from our laboratory indicated higher CORT levels in LP progeny, the current study did not reveal significant differences in CORT levels between NP and LP offspring. This discrepancy may result from variations in measurement techniques, specifically between LC‐MS/MS and immunoassay methods, which may exhibit increased cross‐reactivity [35, 36].

Several studies have highlighted the essential role of 11β‐HSD1 in regulating GC serum levels. The potential knockout of 11β‐HSD1 in various tissues may result in increased CORT levels in low‐protein (LP) models, initiating a negative feedback loop within the HPA axis. The impact of 11β‐HSD1, particularly in the liver, suggests that the reduced 11‐DHC in LP models is due to its degradation by 11β‐HSD1. This investigation proposes that elevated GC levels in LP offspring may lead to sodium retention and hypokalemia, as previously established, closely linked to decreased 11β‐HSD2 levels [37, 38]. Tang et al. [39] study evaluating arterial hypertension in adult offspring after gestational administration of dexamethasone, associated with decreased renal 11β‐HSD2 levels, showed similar effects to those observed in uterine vascular insufficiency and maternal LP intake, and consistent with programming models [40, 41, 42].

Angiotensin‐II (ANG‐II) type 1 and 2 (AT1 and AT2) receptors are expressed in the adrenal and have different regional distribution. The AT1 receptor is predominantly expressed in the granular zone (GZ), while the AT2 receptors are expressed in the adrenal medulla and are responsible for catecholamine release [43, 44]. The current study showed increased type 1 and 2 AT receptors in the adrenal glands of the LP animals at 16 weeks. Previously, authors have shown that in the adrenals from LP offspring subjected to maternal protein restriction, the AT1(b) receptor subtype showed decreased methylation in the gene promoter region, leading to increased expression [45]. This condition is also intimately associated with hypertension development and GC and GR‐enhanced transcription [46]. Bogdarina et al. [47] delved into the study of fetal exposure to maternal GCs, promoting AT1 hypomethylation. Simultaneously, the administration of metyrapone to pregnant rats has fed a LP‐enhanced gene methylation levels in offspring compared to the control. The present study showed increased MR and GR in all adrenal cortical and medulla zones. This finding hints at a potential association between the rise in adrenal AT1 receptors in LP progeny and the increase in GR in GZ. Despite the rise in the AT1 receptor, our study did not find a corresponding increase in plasma aldosterone in these animals. This intriguing discrepancy suggests an alteration in the induction of aldosterone production by ANG‐II receptors. As mentioned above, the ANG‐II has a demonstrated effect on increased plasma Epi and NE at 16‐week‐old LP offspring [23], and AT2 expression is highly associated with TH expression and catecholamine synthesis [48] during prolonged stress [48, 49, 50]. In contrast, our previous study did not detect changes in TH expression in the adrenal glands of 16‐week‐old LP offspring compared to the NP group. These findings, consistent with several others, suggest that stress may not necessarily increase TH mRNA expression. However, this lack of change does not correlate with increased plasma catecholamine. However, the study showed an enhanced medullary area of the adrenal gland in the LP offspring, without differences in the total and cortical area, accompanied by the rise in NeuN, a marker of neuronal cells. We also identified an increase in PCNA in the adrenal gland in 16‐week‐old LP offspring, with intense staining in all cortical and medulla zones. Unfortunately, the study failed to identify phenyl ethanolamine N‐methyltransferase (PNMT) in the adrenal gland, making it impossible to demonstrate whether there is a proportional increase between the types of chromaffin cells. Compared to adrenocortical cells, few studies have demonstrated the capacity of adrenal chromaffin cells to proliferate, suggesting that proliferation may occur according to functional demand. Thus, the depletion of medulla chromaffin granules by reserpine administration leads to increased proliferation of these cells, likely due to the neurogenic proliferative stimulus. Here, the absolute and proportional adrenal and pituitary masses concerning the cortical and medullary areas were decreased in 21 PND LP offspring. This finding, in line with a similar study on animals subjected to food restriction during gestation, underscores the potential long‐term effects of maternal undernutrition on the offspring's endocrine system [51, 52]. These changes promote an adaptive defect in these cells and the cholinergic innervation with functional implications. They also showed increased catecholamine levels, likely not influenced by splenic stimulation once adrenal innervation was completed in the third postnatal week. So, gestational undernutrition underscores the gravity of the potential long‐term effects of maternal undernutrition on the offspring's endocrine system, particularly the RAAS and CORT modulation. Studies in the animal knockout for GR [53, 54] cause defects in the migration of chromaffin cells to the adrenal medulla during the fetal period, altering the adrenal development and catecholamine synthesis in postnatal life. In common among these studies, decreased production and activity of the GC pathway synthesis promotes a decrease or even absence of PNMT in the adrenal medulla [55], justifying the harmful effect of PNMT in the present study. It is valid to assume that these changes in the adrenal development in LP models are influenced by fetal overexposure to GC [56]. Is the increased medullary area and cellularity found in the present study an adaptive effect occurring during intrauterine or postnatal life? This question underscores the crucial need for further studies.

The current study has unveiled a significant increase in the expression of 11β‐HSD1 in the 16‐week‐old LP offspring adrenal, compared to NP progeny. This finding, in conjunction with Shimojo et al. [57] demonstration of the 11β‐HSD1 expressions in the corticomedullary adrenal junction, without change in 11β‐HSD2 after administration of 11β‐HSD inhibitor, led to decreased PNMT without changes in TH, suggesting a direct influence on PNMT expression. Wurtman et al. [58] described that adrenal GC, flowing toward the medulla, exerts a significant influence over the synthesis of PNMT and increases the catecholamine secretion in the adrenal gland of LP offspring [59, 60]. As previously demonstrated, it was associated with increased arterial blood pressure and, as previously observed, increased catecholamine in the kidney and plasma levels that were attenuated by bilateral renal denervation [23]. The observed increase in both receptors in the LP progeny adrenal gland may have significant implications, increasing the steroid release and suggesting that the negative feedback short loop on GC release by the RZ is controlled by the action of GC itself, supporting studies showing desensitization of the adrenal to ACTH by GC's prior administration.

This study confirms the findings of Chong et al. [61] that demonstrate that GR and MR in the adrenal cortical zones control the CORT and aldosterone secretion mediated by ANG‐II stimulation. Specifically, the increased MR in the adrenal GZ acts as an aldosterone production inhibitor. It is worth noting that despite unchanged aldosterone and CORT levels in the present study, our Lab has consistent evidence of disorders in the CNS involved in increased HPA axis activity in LP offspring [11, 12, 29] associated with decreased hippocampal GR in LP animals and dendritic atrophy in CA3, mainly associated with HPA axis hyperactivation [62, 63]. Studies have established that maternal care (licking and cleaning progeny) is a critical factor affecting hippocampal GR expression during the first postnatal weeks by methylation of Exon 17. However, the current study did not identify differences in maternal care between NP and LP groups, suggesting an unexplored mechanism for decreased GR expression in 16‐week‐old LP progeny associated with increased POMC and ACTH in the anterior and intermediary hypophysis. The study also reveals that CRF1 expression is reduced and associated with decreased adenylate cyclase activity. This reduction in CRF1 expression could have significant implications for understanding the regulation of the HPA axis and stress response.

Depending on the duration and type of stressor applied, restoring decreased CRF1 receptor levels after acute or long‐lasting stimuli with transient expression has significant implications. This is followed by an immediate delay in the transcriptional induction and translation of CRF1 mRNA and reduced CRH's binding rate, probably due to receptor degradation [64, 65]. The role of CRF1 receptors in defining sensitivity to stress cannot be overstated. Their availability is crucial in this process [65, 66]. However, an increased CRF1 receptor in the AP of LP offspring was found here without any change in hypothalamic CRH immunostaining. The present data also show increased anterior pituitary GR expression, mainly associated with somatotrophic functions [67]. The increase in POMC and CRF1 receptors in the pituitary of the LP progeny, possibly due to epigenetic mechanisms, is a relevant finding. Nemoto et al. [68] demonstrated that the HPA axis response following food restriction led to a prolonged increase in AP ACTH and CORT, accompanied by POMC and CRF1 mRNA alterations associated with unchanged miR449a and downregulated Crhr1 expressions [69]. The GR positively regulates miR449a expression, and the unchanged miR449a expression was attributed to increased GAS5 ncRNA [70], which is a key insight. AVP's role in ACTH release [71, 72] is a crucial aspect of the hormonal responses in LP progeny. A decreased hypothalamic AVP was previously evidenced [24] associated with reduced ANG‐II‐induced dipsogenic response in 16‐week‐old LP offspring to central administration.

The present findings have revealed significant advances in our understanding of the developmental biology of LP offspring. This prompts the question: Could the increased CORT in LP offspring anticipate the renal and cardiovascular dysfunctions found in low‐protein progeny? The current study showed increased type 1A and 2A 5‐HT receptors in 16‐week‐old LP pituitary lobes. This increase in 5‐HT receptors suggests a potential mechanism for the hyperresponsive state observed in their offspring. The serotonergic neurons are organized in the brainstem's raphe nucleus (RN) with ascending projections innervating the cerebral cortex, hippocampus, hypothalamus, thalamus, basal ganglia, and amygdala, and caudal efferent projections to the cerebellum and descending projections to the spinal cord [73]. The present study found that serotonergic innervations from the RN innervated the hypothalamus, and the pituitary IL may be crucial in modulating POMC and α‐melanocyte‐stimulating hormone (MSH) secretion by enhancing type 1A and 2A 5‐HT receptors expression and increasing prolactin and ACTH secretion but not growth hormone, luteinizing hormone, and thyroid‐stimulating hormone [74, 75, 76, 77, 78]. Furthermore, the current study suggests that in stressful situations, IL pituitary serotonergic nerves raise α‐MSH and β‐endorphin content, and in the PH, oxytocin, a neuropeptide linked to sexual motivation, depression, and anxiety‐simile behavior [79, 80, 81, 82, 83]. Deregulation in serotonin transporters (SERT) leads to a reduction in 5‐HT concentration by extracellular clearance. In a mild stress protocol, an exacerbated response occurred in SERT −/− and SERT +/− animals, causing an increased ACTH secretion and decreased CRF1 receptor expression. So, a decreased SERT concentration in different areas of the CNS may be a sign of stress‐related disorders, which are highly associated with depression development [84, 85]. Here, we consistently show that SERT is expressed in chromaffin cells and is closely linked to 5‐HT and catecholamine secretion. This was further supported by increased catecholamine levels in animals lacking SERT when subjected to restraint stress. The evidence also points to the crucial role SERT plays in this modulation, thereby enhancing the excitability of preganglionic sympathetic neurons in the intermediate‐lateral columns of the thoracic spinal cord.

Brindley et al. [86] revealed that the type 1A 5‐HT receptor promotes inhibition of catecholamine release in stress situations. Therefore, in the present study, we hypothesized that the increased type 1A 5‐HT receptor level in the adrenal medulla associated with enhanced catecholamine secretion in the LP progeny is an attempt to prevent excessive catecholamine release partially. These findings also highlight the engagement of epigenetic mechanisms, broadening the knowledge of gestational low‐protein intake effects on offspring's stress responsiveness and hormonal regulation. Furthermore, the study showed that the 11β‐HSD enzyme modulates stress responsiveness and, at least partially, the hypertension‐CG‐mediated in LP offspring.

In conclusion, the present study demonstrates that maternal protein restriction induces long‐lasting alterations in the structure and function of the HPA axis. This includes upregulation of glucocorticoid and MRs, altered RAAS components signaling, adrenal medullary remodeling, and dysregulated serotonergic control at both pituitary and adrenal levels. The combination of increased CRF1, ACTH/POMC, GR, and serotonergic receptor expression, alongside structural adrenal changes, supports the hypothesis of an intrinsically hyperactive and dysregulated HPA axis in LP offspring. These alterations likely contribute to the heightened stress reactivity, neuroendocrine imbalance, and anxiety behavioral phenotypes previously described in this model [12]. Future investigations should explore receptor functionality, local hormone concentrations, and epigenetic regulators to elucidate the mechanisms underlying these programmed alterations fully.

## Author Contributions

**Vinicius Schiavinatto Mariano:** data curation, investigation, formal analysis, methodology, visualization, writing – original draft. **José Antonio Rocha Gontijo:** conceptualization, formal analysis, methodology, visualization, writing – review and editing. **Patrícia Aline Boer:** conceptualization, formal analysis, funding acquisition, methodology, resources, supervision, visualization, writing – review and editing.

## Conflicts of Interest

The authors declare no conflicts of interest.

## Supporting information

Supplementary Material Mariano et al.

## Data Availability

The data and material are available in https://repositorio.unicamp.br/Resultado/Listar?guid=1738268476906 or https://catalogodeteses.capes.gov.br/catalogo‐teses/#!/.
