# Supplementary material for: Evaluation of the Hypothalamus‐Hypophysis‐Adrenal Axis in Male Rats Programmed by Gestational Protein Restriction
Source: Cell Biochem Funct. 2025 Oct 4;43(10):e70123. doi: 10.1002/cbf.70123 (PMC12495902; doi:10.1002/cbf.70123)
Supplement: Supplementary file 1 — Supplementary Material Mariano et al. [file CBF-43-e70123-s001.docx]

Supplementary Material

Table S1 – Dietic components of both experimental chows of the animals.

| Component | NP diet (%) | LP diet (%) |
| --- | --- | --- |
| Cornstarch | 39.67% | 48.0% |
| Casein (84%) | 20.23% | 7.15% |
| Dextrinizade Starch (90-94%) | 13.05% | 15.9% |
| Sucrose | 10.0% | 12.1% |
| Soybean oil | 7.0% | 7.0% |
| Cellulose microfine (fiber) | 5.0% | 5.0% |
| Mineral mix AIN 93 G | 3.5% | 3.5% |
| Vitamin mix AIN 93 G | 1.0% | 1.0% |
| L-cysteine | 0.3% | 0.1% |
| Choline bitartrate | 0.25% | 0.25% |
| BHT (Butylhydroxitoluol) | 0.0014% | 0.0014% |

| Reagents | Host | Dilution | Supplier | Cat#. |
| --- | --- | --- | --- | --- |
| normal goat serum | - | - | Santa Cruz | sc-2043 |
| normal rabbit serum | - | - | Santa Cruz | sc-2338 |
| Primary antibodies |  |  |  |  |
| 5HT1A | Rabbit | 1:250 (IHC) 1:1000 (WB) | GeneTex | GTX104703 |
| ACTH | Mouse | 1:1000 (IHC and WB) | Santa Cruz | sc-57021 |
| CRHR/CRF1 | Goat | 1:100 (IHC) 1:1000 (WB) | Abcam | **Ab59023** |
| GR | Rabbit | 1:200 (IHC) 1:1000 (WB) | Santa Cruz | **sc-8992** |
| MR | Rabbit | 1:800 (IHC) 1:1000 (WB) | Santa Cruz | **sc-11412** |
| NeuN | Mouse | 1:200 (IHC) 1:1000 (WB) | Millipore | MAB377 |
| 5HT2A | Rabbit | 1:400 (IHC) | GeneTex | GTX37799 |
| 11β-HSD1 | Rabbit | 1:1000 (WB) | Santa Cruz | sc-20175 |
| 11β-HSD2 | Goat | 1:1000 (WB) | Santa Cruz | sc-19263 |
| PCNA | Mouse | 1:1000 | Santa Cruz | sc-25280 |
| Vasopressin | Rabbit | 1:1000 | Millipore | AB1565 |
| TH | Rabbit | 1:1000 | Abcam | Ab112 |
| AT1 | Rabbit | 1:1000 | Santa Cruz | sc-1173 |
| AT2 | Goat | 1:1000 | Santa Cruz | sc-7420 |
| Secondary antibodies |  |  |  |  |
| anti-Mouse IgG HRP | Goat | 1:200 (IHC)  1:8000 (WB) | ThermoFisher | 31430 |
| anti-Goat IgG HRP | Rabbit | 1:200 (IHC) 1:8000 (WB) | ThermoFisher | 31402 |
| anti-rabbit IgG HRP | Goat | 1:200 (IHC) 1:8000 (WB) | Cell Signaling | 7074 |

Table S2 - Antibody and Reagents used.

Table S3 – Anthropometric data of dams of both experimental groups during gestation.

|  | Groups | GD0 | GD7 | GD14 | GD21 | N |
| --- | --- | --- | --- | --- | --- | --- |
| Gestational Weight Gain | *NP (Mean±SD)* | 283,6±24,9 | 313,1±29,2 | 345±35,9 | 366,8±28,8 | 8 |
|  | *LP (Mean±SD)* | 268,2±25,7 | 295,8±29,6 | 320,1±32,8 | 337,3±35 | 11 |
| Gestational Food Intake | *NP (Mean±SD)* | - | 155,27±29,7 | 147,28±20,8 | 141,34±15,9 | 8 |
|  | *LP (Mean±SD)* | - | 149,72±19,7 | 144,85±22,8 | 144,28±30,9 | 11 |


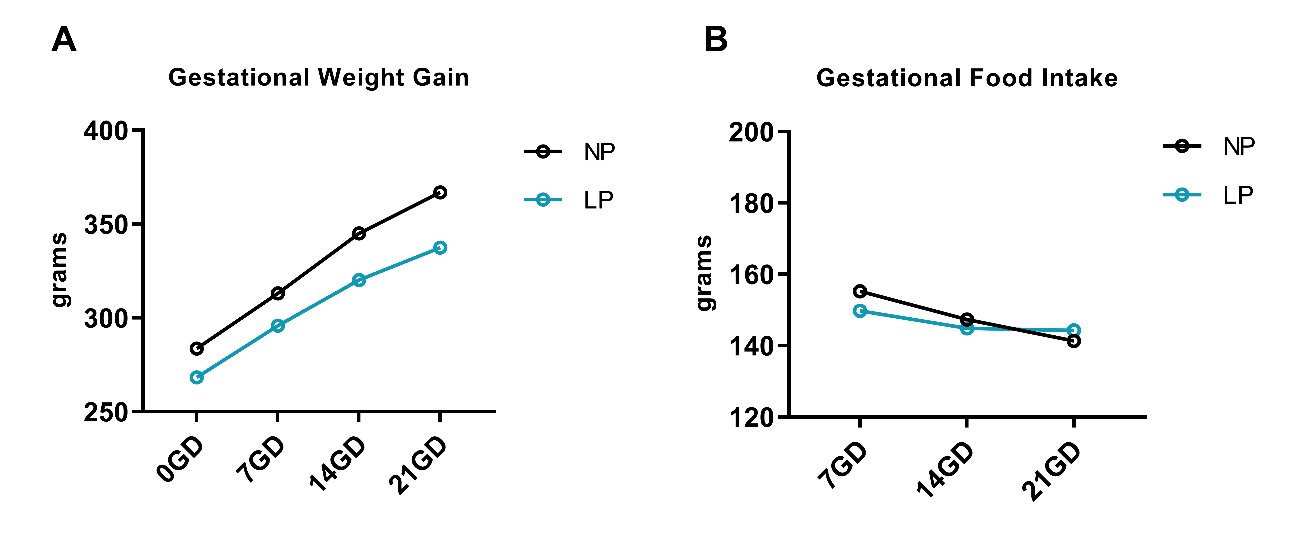


Figure S1 - Anthropometric data of dams of both experimental groups during gestation. (A) Gestational weight gain and (B) food intake of both groups. NP – normal protein diet; LP – low-protein diet. Two-way ANOVA was used for statistical analysis.


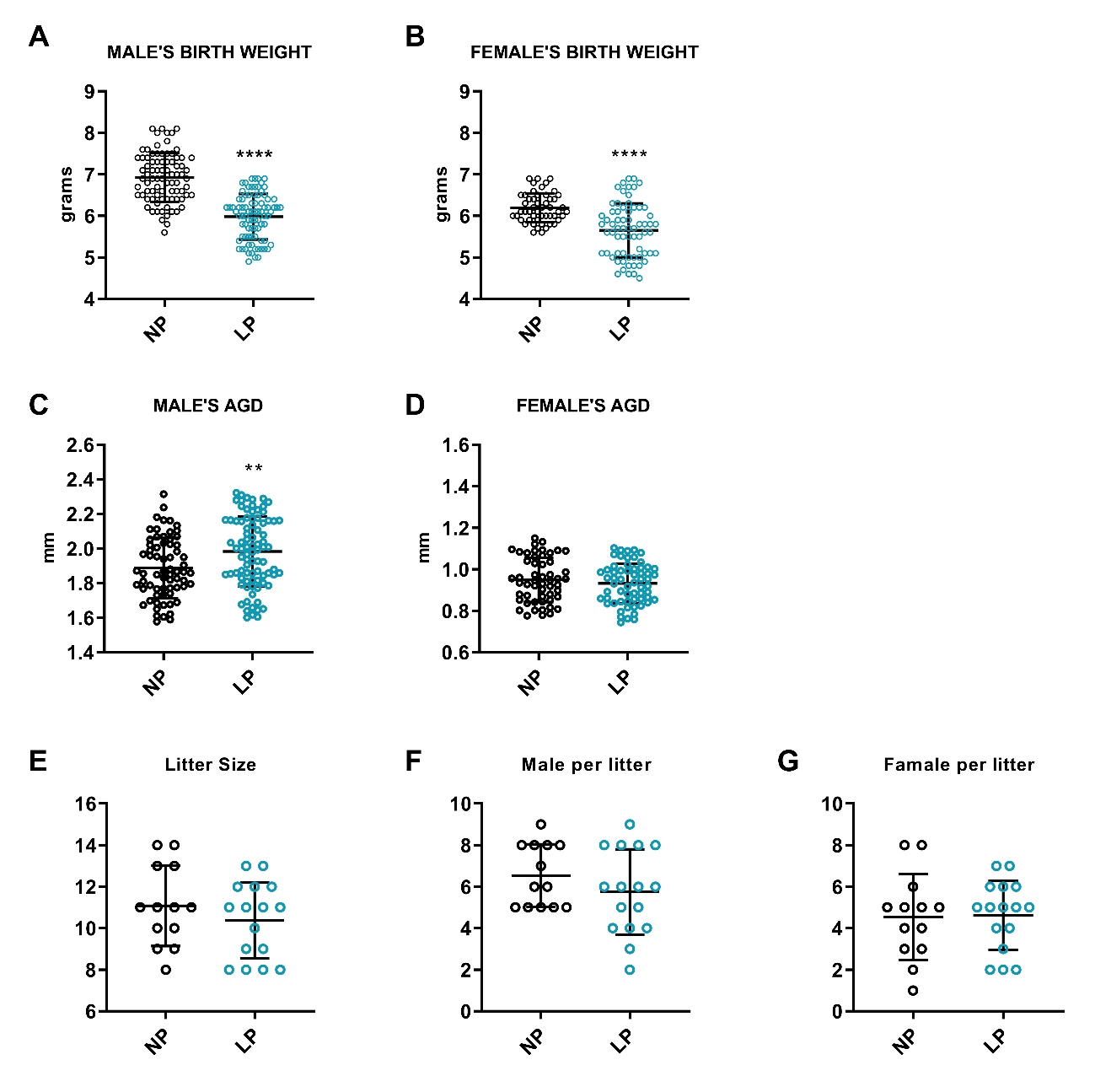


Figure S2 - Anthropometric data of offspring of both experimental groups and sexes. Birth weight of male (A) and female (B) offspring of both groups; ano-genital distance of male (C) and female (D) offspring of both groups. Litter size analyses of total (E), male (F) and female (G) of both experimental groups. NP – normal protein diet; LP – low-protein diet. Student’s t test was used for statistical analysis. (**) <0,01; (****) <0,0001.


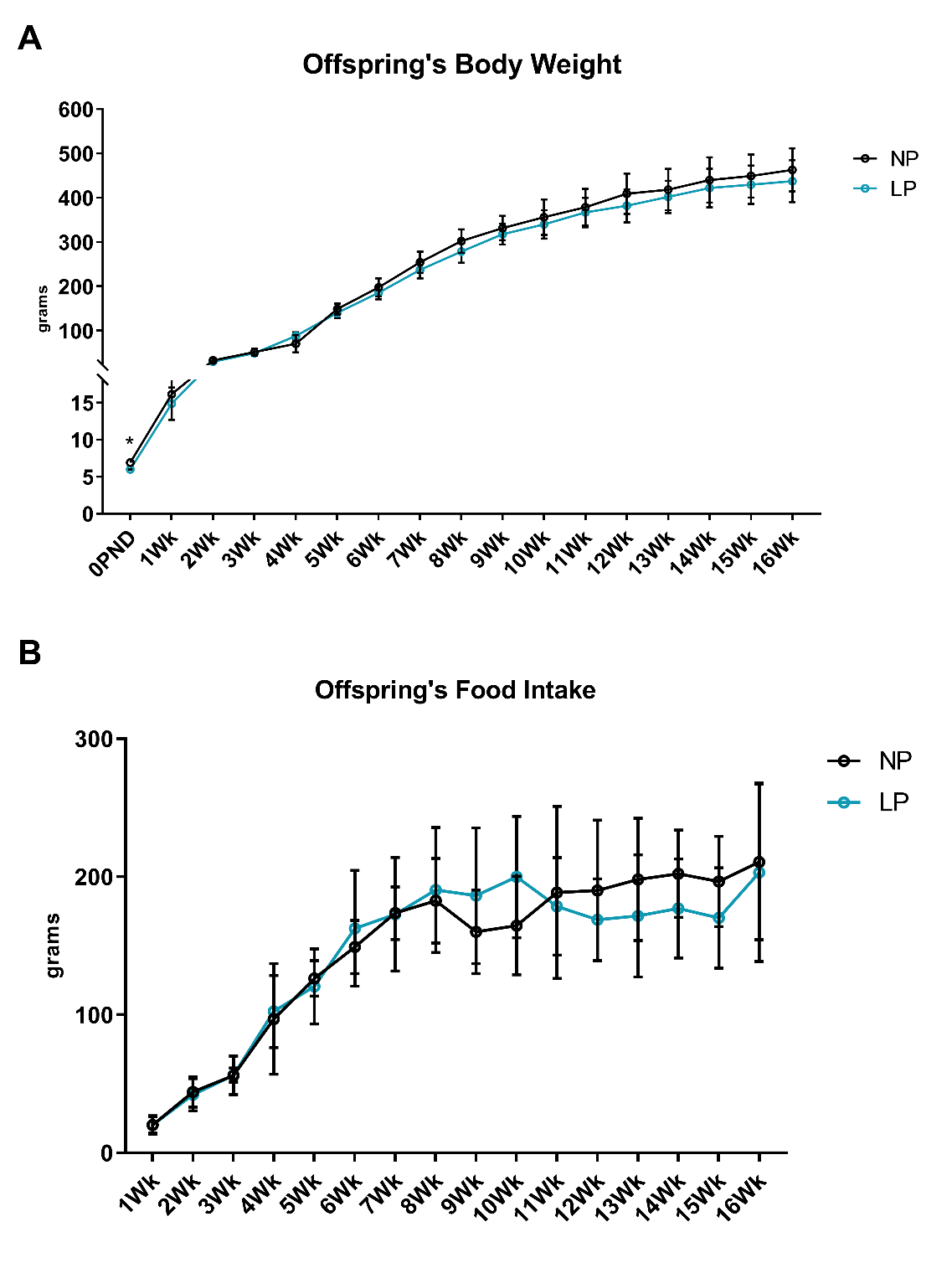


Figure S3 – Postnatal anthropometric data of offspring of both experimental groups. Body weight (A) and food intake (B) of male offspring until 16 week of age. NP – normal protein diet; LP – low-protein diet. Two-way ANOVA was used for statistical analysis.
